# Important aspects of the multimodal perioperative management in gynecology

**DOI:** 10.1007/s00404-025-08043-1

**Published:** 2025-05-14

**Authors:** Susanne Reuter, Babara Schmalfeldt, Sebastian Haas

**Affiliations:** 1 Department of Gynecology, Hamburg-Eppendorf University Medical Center, Martinistraße 52, 20246 Hamburg, Germany; 2 Rudolf-Zenker-Institute for Experimental Surgery, University Medical Center Rostock, Schillingallee 69a, 18057 Rostock, Germany; 3 Department of Anaesthesiology, Intensive Care Medicine and Pain Therapy, University Medical Center Rostock, Schillingallee 35, 18057 Rostock, Germany

**Keywords:** Patient safety, Multimodal perioperative management, Perioperative medication management

## Abstract

Multimodal perioperative management is an integrative, holistic approach to optimizing perioperative patient care. The aim is to accelerate postoperative recovery, minimize complications and increase patient satisfaction. This approach combines various strategies that are tailored to the individual needs of patients. A comprehensive preoperative assessment, in particular preoperative individual risk stratification and perioperative medication management, makes it possible to identify risk factors and take targeted measures. Our overview is intended to provide compact information, particularly for the preoperative setting, and to provide suggestions for practice based on the guideline-oriented summary.

## Introduction

The steady increase in full inpatient gynecological surgery in hospitals is associated with an increase in workload. For example, in 2023, the number of operations on the female reproductive organs alone rose from 576,400 in 2022 to 599,100, and the number of operations on the breast rose from 149,800 in 2022 to 156,00 [1]. In these fast-moving times, it is of increasing importance to firmly implement valid, evidence-based perioperative treatment algorithms in clinical practice to achieve the highest possible level of patient safety. To achieve this goal, it is necessary to renew historical treatment methods with the constant scientific scrutiny of well-known procedures and to introduce this new knowledge into widespread practice as a fixed treatment pathway in the sense of multimodal interdisciplinary perioperative management. For example, several studies have shown that minimally invasive surgical intervention can lead to significantly faster discharge [2, 3], faster convalescence [4], and ultimately also to cost reduction through shorter hospital stays [5]. Taking risk factors into account, more complex laparoscopic gynecological procedures, such as hysterectomy, appear increasingly possible on an outpatient basis with equivalent patient satisfaction and no increase in complications [6]. New treatment concepts are increasingly being developed through interdisciplinary cooperation to further improve convalescence for patients. Corte et al. were able to show that laparoscopic hysterectomy under minimally invasive anesthesia is associated with comparable safety and increased patient satisfaction [7]. We owe one of these first systematic reviews to Kehlet et al. on colorectal carcinoma [8]. However, this work also identified other perioperative risk factors and called for their optimization, ideally already begun preoperatively, such as in the case of malnutrition or alcohol abuse. However, further evidence-based scientific work, such as ending the 6 h preoperative fast [9] and carbohydrate loading [10], has not yet been fully incorporated into our clinical practice. This makes it all the more important to breathe life into perioperative interdisciplinary collaboration to achieve the best possible patient safety and satisfaction. 

## Overview

Perioperative planning should begin with a medical history and assessment of findings. The medical history covers various areas, including personal history, medication history, family history, social history, bleeding history and self-assessment of resilience. In addition, physical examinations, imaging and diagnostic tests (laboratory) are part of the assessment. This is a fundamental process for determining a diagnosis for patients.

The medical history has gained in importance over the centuries. While it does not yet appear in the Corpus Hippocraticum, Rufus of Ephesus dealt with the questioning of patients in the first century. Anamnesis did not play a role in the Middle Ages either, but from the Enlightenment in the seventeenth century it became an integral part of diagnosis with the aim of separating the subjective anamnesis from the objective findings.

**Fig. 1:**
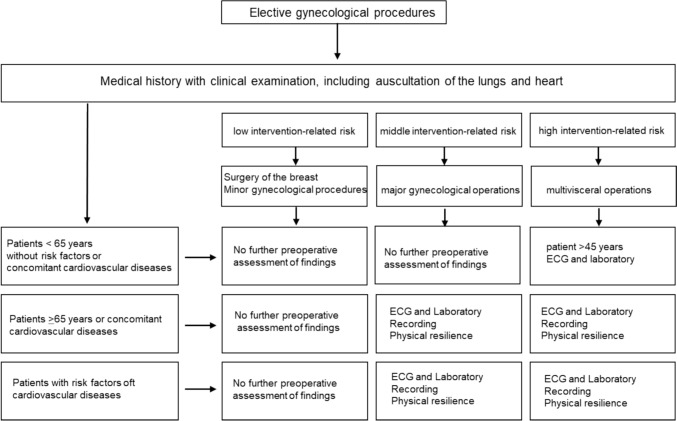
Preoperative risk evaluation of patients before elective gynecological surgery modified according to [12, 16, 17]. It takes into account the type, duration and urgency of the surgical procedure and in most studies refers to a 30-day risk of death from cardiovascular disease, myocardial infarction or stroke. Other risk indices include assessment by ASA, age, concomitant cardiovascular disease, history of stroke and serum sodium, creatinine and hematocrit. Laboratory: Hs-cTn T/I and/or BNP/NT-proBNP; physical capacity: based on the Duke Activity Status Index (DASI) or the ability to climb two flights of stairs

If a clinical picture requiring medical intervention is described, we speak of an indication in this situation. There are four different types of indication. An absolute indication exists if there is a compelling medical reason for an intervention. In the case of acute danger to life, the indication is defined as vital. Emergency indications are when there is a serious acute medical condition that must be treated immediatly in order to save important bodliy functions or life. The relative indication describes an existing reason for treatment, but with recognized treatment alternatives. If there is an indicated non-emergency procedure with a possible scheduled date, an elective indication is assumed. In Germany, 85% of inpatient gynecological operations are elective [1]. With this large proportion of operations, it makes sense to make full use of the pre-inpatient and preoperative intervention options as part of interdisciplinary perioperative management to improve patient safety and preoperative procedures. For example, comprehensive patient information and education on the planned procedure, the anesthesia and the clinical picture should be provided as part of prehabilitation. In this context, prompt information about the surgical procedure and premedication is desirable. To optimize the patient’s condition preoperatively, a balanced diet, abstinence from alcohol and nicotine, physical activity and patient blood management are recommended as standard measures [11–13]. Direct preoperative measures such as thrombosis prophylaxis, short periods of fasting, bowel preparation only depending on the planned procedure, the sparing use or even the omission of sedative premedication, antibiotic prophylaxis and the adjustment of analgesics are addressed in the following text. Physical resilience is a relevant prognostic factor for the risk of postoperative complications in major elective non-cardiac surgery, including pulmonary or cardiac complications and death after 30 days [14]. To assess the individual perioperative risk and possible perioperative complications, the cardiovascular, respiratory and cerebral systems in particular are evaluated. Together with the procedure-related risk, this essential information provides an overall picture. The procedure-related risk takes into account the type, duration and urgency of the procedure and refers to a 30-day risk of death from cardiovascular disease [15], with a classification into low (< 1%), medium (1–5%) and high (> 5%) risk. Other important risk indices are the assessment according to the ASA (American Society of Anesthesiologists) classification, age, concomitant cardiovascular diseases and laboratory values such as serum sodium, creatinine and hematocrit. There are clear recommendations for the scope of preoperative examinations required, adapted to the procedure-related risk, age and risk factors with regard to concomitant cardiovascular diseases (Fig. 1) [12, 16–18]. Biomarkers of the cardio-circulatory system are becoming increasingly important. For example, the German professional societies of anesthesiology and intensive care medicine (DGAI) and surgery (DGCH) are already calling for the preoperative measurement of NTproBNP (N-terminal pro-brain natriuretic peptide) and high-sensitivity troponin in a high-risk surgical constellation to detect previously undetected heart disease [12]. Furthermore, it is even recommended to determine troponin 24 and 48 h after the operation to diagnose perioperative myocardial ischemia at an early stage [12]. These guidelines will certainly present the treating gynecologists/surgeons and anesthesiologists with particular challenges—especially in terms of capacity.

### Prophylaxis of venous thromboembolism

**Fig. 2:**
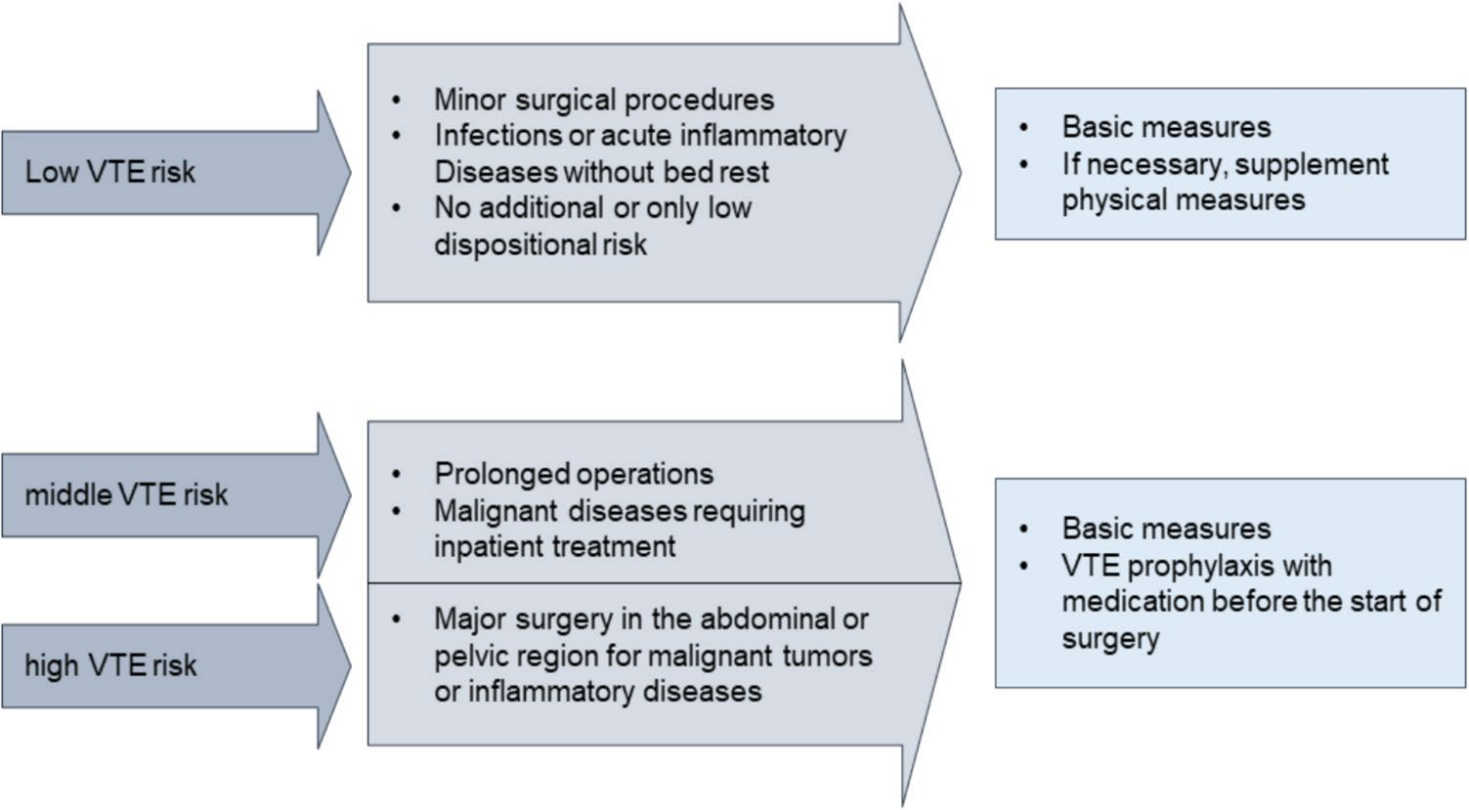
Risk categorization of VTE risk according to AWMF with presentation of the scope of VTE prophylaxis subdivided according to risk groups [20]

Another key aspect of multimodal perioperative management (mPOM) is the prophylaxis of venous thromboembolism (VTE). The indication for VTE prophylaxis should be individualized and risk adapted, as the prevalence of deep vein thrombosis in major gynecological procedures without prophylaxis is between 15 and 40% [19]. The individual risk is made up of exposure and dispositional risk factors. The expositional risk is characterized by the type and extent of a surgical procedure or trauma or an acute illness combined with immobilization. The dispositional risk comprises congenital and acquired personal factors. It is divided into three risk groups (Fig. 2) with a recommendation on the extent and type of VTE prophylaxis [20]. The principles of VTE prophylaxis include basic measures such as early mobilization, physical measures such as medical thrombosis prophylaxis stockings and medicinal measures such as heparins and oral anticoagulants. VTE prophylaxis should be started preoperatively in accordance with the DGAI guidelines for the use of anticoagulants and neuroaxial anesthesia procedures in particular. This measure should be continued 6 h postoperatively for a total duration of 7 days and, in the case of malignancies, 30 days, taking into account and, if necessary, adjusting for renal and liver values and body weight [20].

### Preoperative management of long-term medications

**Fig. 3:**
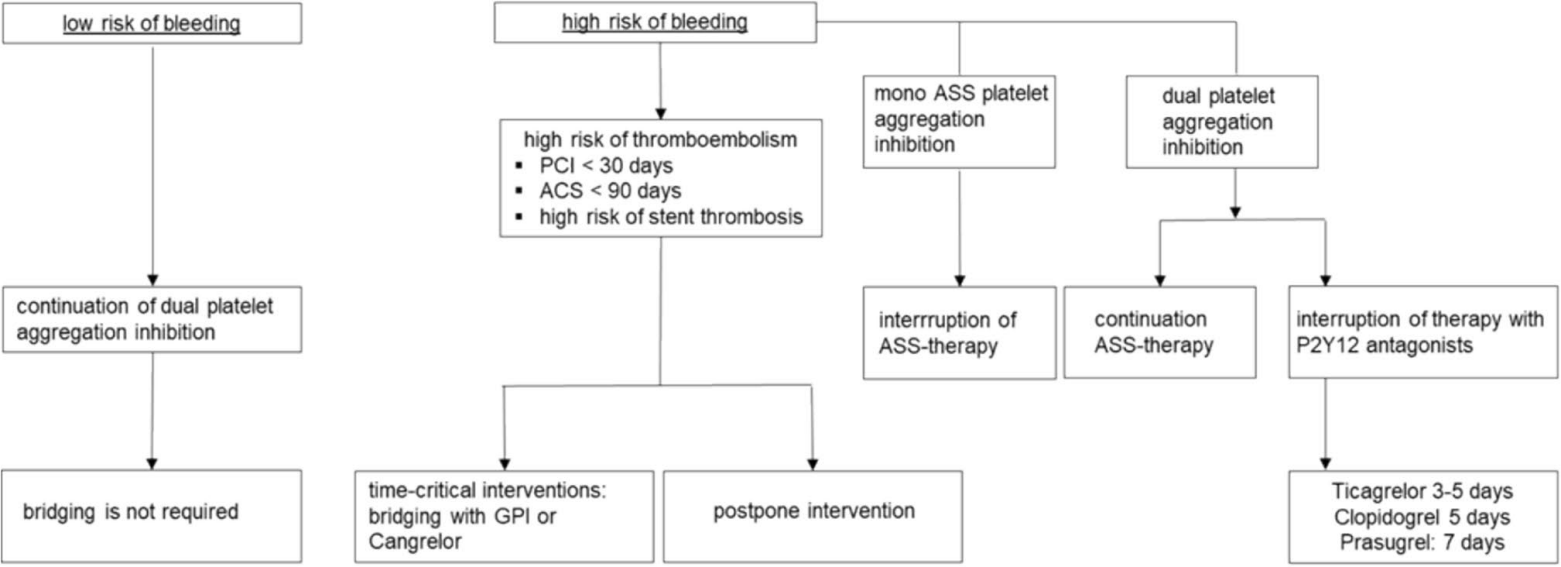
Preoperative recommendation for the treatment of patients with antiplatelet therapy (modified after [12, 16–18]. Bridging: temporary switch to anticoagulants with a short half-life, *GPI* glycoprotein IIb/IIa inhibitors, *PCI* percutaneous coronary intervention, *ACS* acute coronary syndrome

The preoperative management of long-term medication is also of great importance and part of the preoperative evaluation. Various groups of medications, such as anticoagulants, antihypertensives and antidiabetics, require specific adjustments before surgery. However, this adjustment has not yet been conclusively investigated, so that the following recommendations require a critical individual review in each case. In the perioperative use of oral anticoagulants, an overall assessment of the procedure-specific bleeding risk and patient-specific risk factors for thromboembolic complications must be considered individually, as perioperative antithrombotic management has a considerable influence on bleeding complications [12, 21] and mortality [6, 22]. In the case of simple platelet aggregation inhibition with low-dose acetylsalicylic acid, discontinuation of the medication 7 days before surgery is recommended in the case of surgery with a high procedure-related bleeding risk [12, 16–18]. Discontinuation of antiplatelet therapy 7 days before surgery must be very strictly indicated, particularly in patients with cardiac stents, and must be weighed against the sometimes considerable risk of perioperative in-stent stenosis. In case of doubt, a cardiological assessment is essential. Figure 3 shows the detailed procedure for platelet aggregation inhibition depending on the individual bleeding risk. For time-critical, elective surgical procedures, the following preoperative procedure for dual antiplatelet therapy is recommended: If complete discontinuation of P2Y12 antagonists is required for the surgical procedure, current evidence recommends discontinuation of the P2Y12 antagonists ticagrelor for 3–5 days, clopidogrel for 5 days and prasugrel for 7 days prior to a procedure with a high procedure-related bleeding risk. During this time, therapy with ASA is continued. This reduces the risk of major bleeding by 50% without increasing the risk of MACE or death [12, 16–18].Table 1 lists exemplary recommendations for the perioperative management of long-term medication.

**Table 1:** Perioperative management of long-term medication, modified according to [12]

	Recommendation	Note
Antidiabetics		
Sodium–glucose cotransporter 2 inhibitors	Discontinue 24–72 h before surgery	Euglycemic diabetic ketoacidosis
Glucagon-like peptide-1-agonists	Pause on the day of surgery, 7 days break if taken weekly	Caution: gastric emptying disorder with risk of aspiration
Metformin	48 h preoperatively	Caution: lactic acidosis
Sulfonylureas	Pause on the day of surgery	High hypoglycemia risk, caution geriatric patients
Thiazolidindione, insulin sensitizers	Continue long-term medication	Edema, cardiac decompensation
DPP4 inhibitors	Continue long-term medication	
Insulin	Continue long-term medication adapted to blood glucose; on the day of surgery, basic insulin without bolus	Ketoacidosis with relative insulin resistance in post-aggression metabolism
Circulatory drugs		
Beta blockers	Continue long-term medication	Preoperative readjustment not recommended
Ca2 + antagonists	Continue long-term medication	
Diuretics	Pause on the day of surgery	
Angiotensin receptor–neprilysin inhibitors and II-channel Inhibitors	ACE inhibitors: pause on the day of surgery	Transmission in patients with left ventricular dysfunction
ARNI: Angiotensin II receptor blocker and neprilysin inhibitors	Continue long-term medication	
Digitalis glycosides	Continue long-term medication	Narrow therapeutic range
Psychotropic medication		
Tricyclic antidepressants	Continue long-term medication	QT time prolongation
SSRI	Continue long-term medication	With simultaneous administration of serotomimetic substances before serotonin syndrome
Lithium	Break 72 h before surgery	
Neuroleptics	Continue long-term medication	QT time prolongation Α1 adrenergic antagonistic effect
Anticonvulsants	Continue long-term medication	Interaction with cytochrome P450, possibly increased need for muscle relaxants, hypnotics
Anti-parkinsonian drugs	Continue long-term medication	With administration of dopamine antagonists (MCP)
Corticosteroids, analgesics and statins		
Corticosteroids	Continue long-term medication (> 5 days)	
Analgetics	Continue long-term medication	Inclusion in OP requirements Transdermal systems
Co-analgetics	Continue long-term medication	QT time prolongation, Α1 adrenergic antagonistic effect
Statins	Continue long-term medication	Anti-inflammatory, plaque-stabilizing effect

### Perioperative intravenous antibiotic prophylaxis (PAP)

Postoperative wound infections (surgical site infection, SSI) account for almost 25% of all nosocomial infections occurring in Germany and can increase morbidity and mortality [23]. Ideally, prophylactic administration is recommended within 1 h preoperatively, but at the latest 30 min before the incision. For all procedures lasting less than 3 h, a single dose of the antibiotic (so-called single shot) is usually sufficient. A second dose should be given depending on the half-life or in the event of major blood loss (> 1.5 l) during the operation. The postoperative continuation of PAP is not recommended, as it increases the SSI rate during visceral surgery, but increases resistance and toxicity (renal failure), C. difficile infections and costs [23]. 

### Preoperative bowel preparation

The assessment of the need for bowel preparation in procedures such as deep infiltrating rectal endometriosis or in gynecologic carcinoma surgery with colorectal resection has undergone a change. Purely mechanical bowel preparation (MBP) is no longer recommended [24]. If preoperative bowel preparation is performed prior to colorectal surgery, it should be a combined mechanical and oral antibiotic bowel preparation. A recent Cochrane review shows that a combined mechanical and oral antibiotic bowel preparation compared to MBP alone leads to a reduction in the risk of infection in the surgical site (RR 0.56; 95% CI 0.42–0.74) and anastomotic insufficiencies (RR 0.60; 95% CI 0.39–0.99) [24]. A broad-spectrum oral antibiotic, such as rifaximin at a dose of 200 mg every 8 h, is recommended for use in oral antibiotic bowel preparation, starting 24 h preoperatively [24]. The advantage of this active ingredient is the lack of absorption and the absence of systemic side effects, as well as a high concentration in the intestine, which exceeds the minimum inhibitory concentration of sensitive pathogens many times over. The antibiotic is effective against all germs relevant to intestinal infections, including *Chlostridium difficile* and enteroaggregative *Eschericha coli*. The influence on the physiological intestinal flora also returns to normal 1–2 weeks after use [25].

### Prophylaxis against postoperative nausea and vomiting (PONV)

**Fig. 4:**
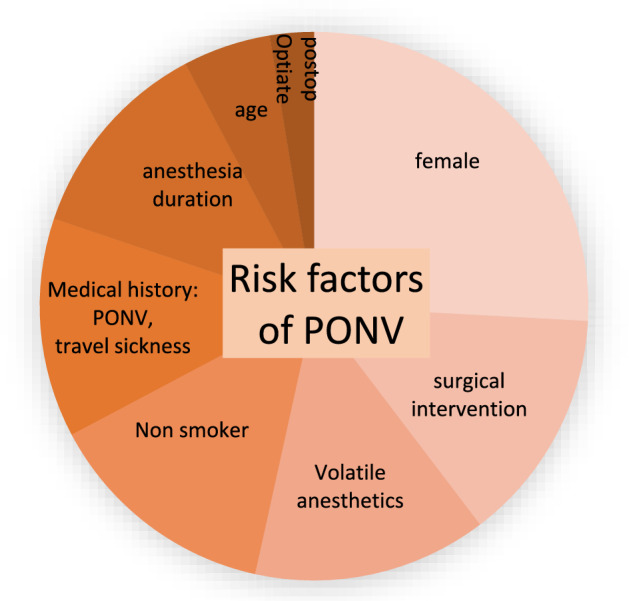
Illustration of risk factors for the development of PONV in adults

The incidence of postoperative nausea and vomiting (PONV) after general anesthesia using inhalation anesthetics without PONV prophylaxis is described in the literature as at least 30% [26]. We are aware of several risk factors, as shown in Fig. 4. This shows that at least two significant risk factors are always present in surgical gynecology and that combination prophylaxis and multimodal antiemetic therapy are required [27].

The following aspects must always be taken into account for PONV prophylaxis [12, 27].

- Dexamethasone, droperidol and ondansetron have been shown to have comparable antiemetic efficacy with a relative reduction in the risk of PONV of around 26%.
- The effects of a combination of these antiemetic measures (dexamethasone, droperidol, ondansetron and total intravenous anesthesia (TIVA, propofol instead of volatile anesthetics) are additive.
- The PONV-protective effect of a TIVA is only effective intraoperatively and cannot be “made up for” in the recovery room or on the ward. TIVA should therefore be prioritized over the administration of antiemetics, especially in cases of above-average risk. Nevertheless, it must be noted that even the performance of a TIVA does not rule out the development of PONV.
- There is currently no evidence that a specific antiemetic is particularly effective for a specific patient or a specific operation.

The higher the underlying PONV risk, the more components are required from the available antiemetic portfolio to achieve a required PONV risk of less than 20% [26, 27]. Rapid treatment is also indicated if PONV occurs, as there is at least a 65% probability that the symptoms will persist or recur over 24 h [28, 29]. For reasons of practicability, the same dosages are recommended for treatment as for prophylaxis [30]. Drugs that are associated with a slower onset of action (e.g., dexamethasone, scopolamine) should not be used as a single intervention, but at best in combination with a fast-acting substance as part of the treatment. If PONV occurs despite prophylaxis, a substance from a different pharmacological group should be used [26]. A variety of substances from different drug groups are available for PONV prophylaxis. An example recommendation for PONV prophylaxis is shown in Table 2. Stimulation of the acupuncture point P6 on the wrist has been confirmed to be effective as an adjuvant and non-drug measure for PONV prophylaxis compared to placebo and is recommended as an additive component in the multimodal treatment concept [23].

**Table 2:** Exemplary recommendation for PONV prophylaxis [27]

Adults at risk of PONV: preferably propofol-based TIVA		
Dexamethasone	4–8 mg i.v	After anesthesia induction
Granisetron	1–3 mg i.v	Before anesthesia induction
Additionally for high PONV risk		
Aprepitant	80 mg p.o	As premedication on call for surgery
Droperidol	0.625–1.25 mg i.v	30–60 min before anesthesia induction

### Cost reduction

The implementation of multimodal perioperative management was also described by several working groups as being associated with reducing treatment costs. The fast-track concept, the introduction and implementation of the ERAS concept and the concept of the extended recovery room of the PACU24 are pioneering in this respect and characterized by interdisciplinarity. The implementation of these concepts has resulted in shorter inpatient stays, a reduction in perioperative complications and, as demonstrated by Kastrup et al. by the analysis of administrative data of more than 50.000 patients, in a reduction in costs [31–37]. The main objective of the PACU24 is the interdisciplinary and interprofessional implementation of the building blocks of multimodal perioperative management, again with the primary treatment goal of reducing postoperative complications and increasing patient safety.

## Conclusion

Well-networked collaboration between the various specialties improves comprehensive patient care and leads to greater patient safety and satisfaction. This includes an individual risk assessment to identify individual patient needs and develop customized treatment plans. Comprehensive patient education throughout the entire perioperative process, including preparation for the procedure and aftercare, reduces anxiety and increases patient compliance. Perioperative risks can be reduced by implementing standardized multimodal measures, such as perioperative prophylaxis of venous thromboembolism, nutritional management, preoperative management of long-term medication, administration of perioperative intravenous antibiotic prophylaxis and multimodal prophylaxis of postoperative nausea and vomiting. Well-connected perioperative interdisciplinary management might even result in a positive impact on healthcare costs. We see this review article on multimodal perioperative management in gynecology as an inspiration to consistently implement this in practice with all its advantages.

## Data Availability

No datasets were generated or analysed during the current study.
